# Perioperative Outcomes After Robotic Inguinal Hernia Repair in Patients With BMI ≥ 35 kg/m2: A Retrospective Cohort Study

**DOI:** 10.7759/cureus.111238

**Published:** 2026-06-21

**Authors:** Michelle Lee, Alexandra Nguyen, Aldin Malkoc, Amanda Daoud, Harpreet Gill, Sunal Patel, Deron Tessier, Carie McVay

**Affiliations:** 1 Surgery, Kaiser Permanente, Fontana, USA

**Keywords:** comorbid obesity, inguinal hernia repair, obesity, robotic hernia, surgical repair of hernia

## Abstract

Introduction: Although robotic inguinal hernia repair (RIHR) is increasingly utilized, limited data exist regarding its safety in patients with body mass index (BMI) ≥ 35 kg/m^2^. This study compares perioperative outcomes and 24-month recurrence rates following RIHR between patients with BMI < 35 kg/m^2^ and BMI ≥ 35 kg/m^2^.

Methods: A retrospective review was conducted for adult patients undergoing RIHR between February 2019 and July 2022 at a single quaternary care center. Patients were stratified into BMI < 35 kg/m^2^ and BMI ≥ 35 kg/m^2^. Demographics, comorbidities, perioperative variables, postoperative complications, and recurrence rates within two years were analyzed. Propensity score matching was performed for age, sex, and hernia type.

Results: A total of 106 patients underwent RIHR, including 90 patients (84.9%) with BMI < 35 kg/m^2^ and 16 patients (15.1%) with BMI ≥ 35 kg/m^2^. No statistically significant differences were observed in operative time, estimated blood loss, or postoperative complications. Rates of hernia recurrence were comparable between the two groups at 24 months.

Conclusion: Our study shows that RIHR has comparable perioperative outcomes and 24-month recurrence rates in patients with BMI < 35 kg/m^2^ and BMI ≥ 35 kg/m^2^. These findings suggest that elevated BMI alone should not preclude RIHR.

## Introduction

Inguinal hernia repair is among the most performed general surgical procedures worldwide, with lifetime risks approaching 27% in men and 6% in women [1]. Surgical management has significantly evolved over the past century, progressing from traditional tissue-based tension repairs, including Bassini and Shouldice techniques, to modern tension-free mesh-based repairs following the introduction of the Lichtenstein technique [2]. To date, tension-free mesh repair has become the standard open approach because of lower recurrence rates and improved postoperative outcomes compared with traditional tissue repairs [3].

With the advancement of minimally invasive surgery, laparoscopic inguinal hernia repair (LIHR) emerged as an alternative to open inguinal hernia repair (OIHR), particularly for bilateral and recurrent hernias. Contemporary international guidelines support both open and laparoscopic approaches, although ongoing debate remains regarding operative cost, learning curve, postoperative pain, recovery time, and recurrence outcomes between techniques [4]. Meta-analyses have demonstrated that LIHR is associated with faster recovery and lower chronic groin pain, while OIHR remains widely performed due to technical familiarity, shorter operative times in select patients, and lower procedural cost [5].

More recently, robotic inguinal hernia repair (RIHR) has gained acceptance as robotic platforms offer enhanced three-dimensional visualization, improved ergonomics, and wrist instrumentation [6]. Given this, robotic surgery may facilitate minimally invasive repair in technically challenging patients. However, the robotic approach for inguinal hernia repair remains controversial because of increased operative cost and limited long-term comparative data relative to open and conventional laparoscopic techniques [7].

Obesity continues to represent a major public health concern worldwide, with current estimates indicating that over 650 million adults globally are classified as obese [8]. Patients with elevated BMI frequently present with greater perioperative complexity, including increased abdominal wall thickness, difficult exposure, higher anesthetic risk, and greater comorbidity burden. Particularly, BMI < 35 kg/m^2^ has been identified as the optimal critical value for perioperative safety in inguinal hernia repairs [9]. Prior studies evaluating RIHR in obese patients have primarily used BMI ≥ 30 kg/m^2^ as the obesity threshold and generally demonstrated comparable perioperative outcomes relative to lower BMI cohorts [10-12]. However, there exists limited data specifically evaluating outcomes in patients with BMI ≥ 35 kg/m^2^, a subgroup often perceived to be at a higher technical and perioperative risk during minimally invasive surgery.

Therefore, this study aimed to evaluate perioperative outcomes and 24-month recurrence rates following RIHR in patients with BMI < 35 kg/m^2^ compared to patients with BMI ≥ 35 kg/m^2^ at a single quaternary referral center.

## Materials and methods

A retrospective cohort review was performed of adult patients (≥ 18 years old) who underwent RIHR between February 1, 2019, and July 31, 2022, at a single quaternary care center.

Diagnosis of inguinal hernia was primarily clinical, with cross-sectional imaging or ultrasonography selectively obtained in patients with equivocal exam findings. Eligible cases included unilateral or bilateral inguinal hernias, with or without concomitant ventral or umbilical hernia repair. Patients undergoing concomitant ventral or umbilical hernia repair were included, given that these procedures were performed during the same operative session. To minimize confounding variables, hernia type was incorporated into the propensity score matching model. All procedures were performed using the Da Vinci Xi robotic platform (Intuitive Surgical, Sunnyvale, CA). A transabdominal preperitoneal approach with synthetic mesh was utilized in all cases with general anesthesia.

Patients with documented preoperative BMI and available operative and postoperative follow-up data up to 24 months were included in the study. Patients were excluded if they underwent open or laparoscopic inguinal hernia repair, had incomplete operative records, lacked documented BMI, or did not present to their follow-up appointments. The data collected included patient age, gender, medical comorbidities, operative time (time from incision to last suture), postoperative complications, and recurrence rates. Medical comorbidities included hypertension, coronary artery disease, diabetes mellitus type two, abdominal aortic aneurysm, smoking, hyperlipidemia, obstructive sleep apnea, and atrial fibrillation.

Primary outcomes included postoperative complications and hernia recurrence within 24 months. Hernia recurrence was identified by physical exam or with appropriate imaging for equivocal exams. Postoperative complications were defined as any adverse postoperative event, including seroma, urinary complications, surgical site infections, or pulmonary embolism. Hernia recurrence was analyzed as an independent endpoint. Data gathering and analysis were conducted according to the Kaiser Permanente Institutional Review Board.

Data were analyzed using the Statistical Product and Service Solutions (SPSS, version 27.0; IBM SPSS Statistics for Windows, Armonk, NY) software. Continuous data were presented according to the means with standard deviation, and categorical data were presented with frequencies and proportions. A T-test was utilized for continuous data, and where appropriate, nonparametric continuous data were analyzed using Mann-Whitney U tests. Univariate analyses were performed using chi-squared for categorical data. For analysis, included patients were stratified into two groups based on BMI: BMI < 35 kg/m^2^ and BMI ≥ 35 kg/m^2^. Propensity score-matched analysis was performed in a 1:1 nearest-neighbor fashion without replacement using a caliper width of 0.01. Matching variables included age, sex, and type of hernia because these variables were considered most clinically relevant to operative complexity and recurrence risk. Standardized mean differences were assessed following matching to evaluate covariate balance. Unless otherwise indicated, p-value < 0.05 was statistically significant.

## Results

Of 106 patients, 90 patients (84.9%) had BMI < 35 kg/m^2^ and 16 patients (15.1%) had BMI ≥ 35 kg/m^2^. Mean ages were 61.4 ± 13.8 years in the BMI < 35 kg/m^2^ group and 54.2 ± 14.3 years in the BMI ≥ 35 kg/m^2^ group (p=0.08).

Gender distribution did not significantly differ between groups (p=0.35). Smoking prevalence was higher in the BMI ≥ 35 kg/m^2^ group (3/16 patients, 18.8%) compared to the BMI < 35 kg/m^2^ group (4/90 patients, 4.4%) (p=0.03). However, other comorbidities, such as hypertension, diabetes mellitus, coronary artery disease, abdominal aortic aneurysm, hyperlipidemia, obstructive sleep apnea, and history of atrial fibrillation, were comparable between groups (p>0.05). Hernia types, including unilateral or bilateral occurrence, and concomitant umbilical or ventral hernias, were similar between groups (p=0.51). There were no statistical differences in operative time between the BMI < 35 kg/m^2^ group (185.76 ± 65.45 minutes) and the BMI ≥ 35 kg/m^2^ group (205.77 ± 58.29 minutes) (p=0.23). Postoperative complications occurred in 5/106 patients (4.7%) in the BMI < 35 kg/m^2^ group only, but differences were not statistically significant (p=0.14). These findings are summarized in Table 1.

**Table 1:** Baseline patient characteristics with total patient population and propensity matched

Demographics	Total Patient Population			Propensity-Matched Analysis for Age, Gender, and Type of Hernia		
	Body Mass Index Under 35 kg/m², N = 90	Body Mass Index Over 35 kg/m², N = 16	P	Body Mass Index Under 35 kg/m², N = 12	Body Mass Index Over 35 kg/m², N = 12	P
Age	61.42 +/- 13.82	54.18 +/-14.33	0.076	49.33 +/-15.43	53.50 +/-12.87	0.480
Gender			0.354			0.615
Male	76 (84%)	12 (75%)		10 (83%)	9 (75%)	
Female	14 (16%)	4 (25%)		2 (17%)	3 (25%)	
Operative Time (minutes)	185.76 +/- 65.45	205.77 +/- 58.29	0.228	172.08 +/- 65.41	215.08 +/- 58.05	0.103
Blood Loss (mL)	11.46 +/- 13.72	13.43 +/- 23.43	0.747	14.10 +/- 15.34	15.83 +/- 26.86	0.854
Comorbid Conditions						
Hypertension (%)	29 (32%)	9 (56%)	0.065	1 (8%)	6 (50%)	0.025
Coronary Artery Disease (%)	6 (7%)	0 (0%)	0.288	0 (0%)	0 (0%)	N/A
Diabetes Mellitus (%)	11 (12%)	2 (13%)	0.975	1 (8%)	2 (17%)	0.537
Abdominal Aortic Aneurysm (%)	2 (2%)	1 (6%)	0.371	1 (8%)	0 (0%)	0.307
Smoking (%)	4 (4%)	3 (19%)	0.034	0 (0%)	3 (25%)	0.064
Hyperlipidemia (%)	34 (38%)	4 (25%)	0.326	6 (50%)	1 (8%)	0.025
Obstructive Sleep Apnea (%)	15 (17%)	0 (0%)	0.078	0 (0%)	0 (0%)	N/A
History of Atrial Fibrillation (%)	2 (2%)	0 (0%)	0.547	0 (0%)	0 (0%)	N/A
Total Comorbidities	1.18 +/-1.12	1.18 +/-1.22	0.997	0.83 +/-0.71	1.0 +/- 0.85	0.610
Complications (%)	5 (6%)	0 (0%)	0.140	1 (8%)	0 (0%)	0.307
Types of Hernia			0.514			0.483
Right Inguinal (%)	26 (29%)	4 (25%)		7 (58%)	3 (25%)	
Left Inguinal (%)	7 (8%)	0 (0%)		0 (0%)	0 (0%)	
Bilateral Inguinal (%)	35 (38%)	5 (32%)		2 (16%)	4 (33%)	
Umbilical and Single-Sided Inguinal	3 (3%)	2 (12%)		0 (0%)	0 (0%)	
Umbilical and Bilateral Inguinal	5 (6%)	1 (6%)		1 (8%)	1 (8%)	
Ventral and Single-Sided Inguinal	6 (7%)	1 (6%)		0 (0%)	1 (8%)	
Ventral and Bilateral Inguinal	8 (9%)	3 (19%)		2 (16%)	3 (25%)	

**Figure 1:**
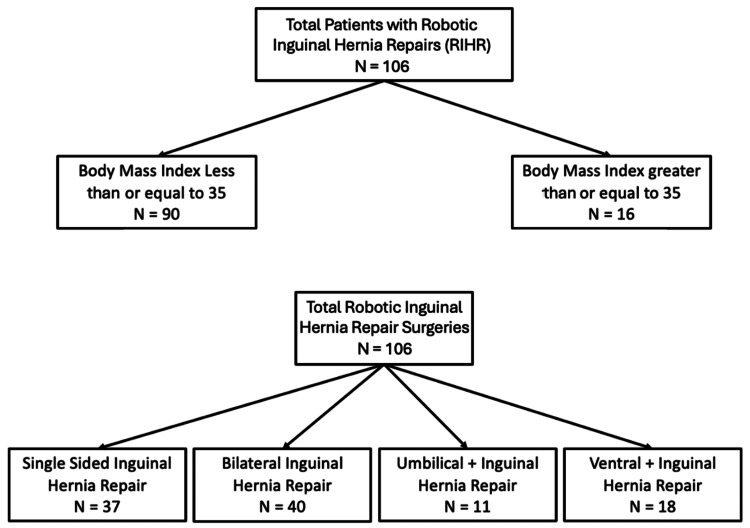
CONSORT diagram detailing the patients who underwent inguinal hernia repair CONSORT: CONsolidated Standards Of Reporting Trials

Most hernia types were isolated inguinal hernias without other concurrent hernias, as demonstrated in Figure 1. Concomitant umbilical or ventral hernias were noted in 27% of patients.

Hernia recurrence occurred in six patients in only the BMI < 35 kg/m^2^ cohort, with half the recurrences (n=3) observed within six months and the other half (n=3) observed at 24 months. Clinical variables from both Group A and Group B, including age, operative time, comorbidities, complications, body mass index, and hernia type, were analyzed, and none were found to have a statistically significant association with hernia recurrence (p≥0.05). However, because only six occurrence events were observed, the multivariate regression analysis was underpowered. Therefore, recurrence analyses should be interpreted cautiously, rather than definitively.

## Discussion

In this retrospective single-center cohort study, we did not identify significant differences in operative time, estimated blood loss, postoperative complications, or 24-month recurrence rates between patients with BMI < 35 kg/m^2^ and BMI ≥ 35 kg/m^2^ undergoing RIHR. Although the study was underpowered to detect small differences due to the limited number of patients in the BMI ≥ 35 kg/m^2^ cohort, these findings suggest that RIHR is technically feasible in selected patients with elevated BMI.

Previous studies predominantly examining patients with BMI ≥ 30 kg/m^2^ demonstrated comparable perioperative and postoperative outcomes with those of lower BMI cohorts. Chinn et al. reported that postoperative outcomes and recurrence of inguinal hernia after undergoing RIHR remain comparable across BMI categories (BMI < 25 kg/m^2^, 25-29.9 kg/m^2^, ≥ 30 kg/m^2^) [11]. In a multi-institutional study, Kolachalam et al. observed no difference in intraoperative or postoperative complications between BMI ≥ 30 kg/m^2^ and BMI < 30 kg/m^2^ patients undergoing RIHR [12]. Recently, a 2025 National Inpatient Sample (NIS) analysis comparing RIHR and LIHR in patients with BMI ≥ 30 kg/m^2^ reported that RIHR was associated with a significantly lower risk of complications and shorter hospital stays, albeit at a higher cost [13]. Our findings suggest comparable outcomes within the extended BMI ≥ 35 kg/m^2^ cohort from the prior studies and support a robotic approach as it may confer comparable benefits in morbidly obese patients without elevated peri-operative or postoperative risk. While cost analyses were beyond the scope of our study, the similarity in clinical outcomes observed in our study suggests that RIHR offers benefits in patients with BMI ≥ 35 kg/m^2^.

The primary strengths of this study include a two-year follow-up on recurrence and the use of propensity score matching to adjust for confounding factors in BMI categorization, comorbidities, and hernia type. However, the study remains limited by its retrospective design, which can introduce selection bias regarding which patients underwent robotic rather than open or laparoscopic repair. Because robotic approach selection was surgeon-dependent, unmeasured confounders may have influenced outcomes. The inclusion of concomitant ventral and umbilical hernia repairs also introduced procedural heterogeneity that may have affected operative duration and perioperative outcomes. Additionally, the absence of statistically significant differences should not be interpreted as equivalence between BMI groups. The study included only 16 patients in the BMI ≥ 35 kg/m^2^ cohort and six total recurrence events, substantially limiting statistical power and increasing the risk of type II error.

## Conclusions

In this retrospective single-center study, RIHR in patients with BMI ≥ 35 kg/m^2^ demonstrated perioperative outcomes and short-term recurrence rates comparable to those observed in patients with BMI < 35 kg/m^2^. While prior studies have often considered patients with a BMI ≥ 30 kg/m^2^, our study specifically evaluates outcomes in patients with a higher BMI, which has been a historically perceived as a greater risk. Given the small sample size and limited statistical power, these findings should be interpreted cautiously. Nevertheless, the study supports the technical feasibility of RIHR in selected patients with elevated BMI and highlights the need for larger prospective multicenter studies evaluating long-term outcomes and comparative effectiveness between robotic, laparoscopic, and open approaches.

## References

[1] Bradley EM, Schlosser KA, Matheny ME, Pierce RA (2026). Evolving inguinal hernia repair practice at the Veterans Health Administration. JAMA Surg.

[2] Eltair M, Hajibandeh S, Hajibandeh S (2019). Meta-analysis of laparoscopic groin hernia repair with or without mesh fixation. Int J Surg.

[3] Fitzgibbons RJ Jr, Forse RA (2015). Groin hernias in adults. N Engl J Med.

[4] Lockhart K, Dunn D, Teo S, Ng JY, Dhillon M, Teo E, van Driel ML (2018). Mesh versus non-mesh for inguinal and femoral hernia repair. Cochrane Database Syst Rev.

[5] Aiolfi A, Cavalli M, Micheletto G (2019). Primary inguinal hernia: systematic review and Bayesian network meta-analysis comparing open, laparoscopic transabdominal preperitoneal, totally extraperitoneal, and robotic preperitoneal repair. Hernia.

[6] Tatarian T, Nie L, McPartland C (2021). Comparative perioperative and 5-year outcomes of robotic and laparoscopic or open inguinal hernia repair: a study of 153,727 patients in the state of New York. Surg Endosc.

[7] Glasgow RE, Mulvihill SJ, Pettit JC (2021). Value analysis of methods of inguinal hernia repair. Ann Surg.

[8] Abad-Jiménez Z, Vezza T (2025). Obesity: a global health challenge demanding urgent action. Biomedicines.

[9] Hajibandeh S, Hajibandeh S, Harries K, Lewis WG, Egan RJ (2025). Critical values for body mass index related to morbidity in high-volume low-complexity general surgery: a systematic review and meta-analysis. Ann R Coll Surg Engl.

[10] Kudsi OY, Bou-Ayash N, Gokcal F (2022). Comparison of perioperative outcomes between non-obese and obese patients undergoing robotic inguinal hernia repair: a propensity score matching analysis. Hernia.

[11] Chinn J, Tellez R, Huy B (2022). Comparison of BMI on operative time and complications of robotic inguinal hernia repair at a VA medical center. Surg Endosc.

[12] Kolachalam R, Dickens E, D'Amico L, Richardson C, Rabaza J, Gamagami R, Gonzalez A (2018). Early outcomes of robotic-assisted inguinal hernia repair in obese patients: a multi-institutional, retrospective study. Surg Endosc.

[13] Pai HJ, Hsieh CC (2025). In-hospital outcomes of robotic versus laparoscopic inguinal hernia repair in obese patients: a national inpatient sample analysis 2005-2020. Hernia.

